# A Novel Inhibitor of Mammalian Triosephosphate Isomerase Found by an *In Silico* Approach

**DOI:** 10.1155/2014/469125

**Published:** 2014-03-23

**Authors:** Lorraine Marsh, Kaushal Shah

**Affiliations:** Department of Biology, Long Island University, 1 University Plaza, Brooklyn, NY 11201, USA

## Abstract

Triosephosphate isomerase (TIM) is an essential, highly conserved component of glycolysis. Tumors are often dependent on glycolysis for energy and metabolite production (the Warburg effect). Glycolysis inhibitors thus show promise as cancer treatments. TIM inhibition, unlike inhibition of other glycolysis enzymes, also produces toxic methylglyoxal targeted to regions of high glycolysis, an effect that might also be therapeutically useful. Thus TIM is an attractive drug target. A total of 338,562 lead-like molecules were analyzed computationally to find TIM inhibitors by an efficient “double screen” approach. The first fragment-sized compounds were studied using structure-based virtual screening to identify binding motifs for mammalian TIM. Subsequently, larger compounds, filtered to meet the binding criteria developed in the first analysis, were ranked using a second round of structure-based virtual screening. A compound was found that inhibited mammalian TIM *in vitro* in the micromolar range. Docking and molecular dynamics (MD) suggested that the inhibitor made hydrogen bond contacts with TIM catalytic residues. In addition, hydrophobic contacts were made throughout the binding site. All predicted inhibitor-TIM interactions involved TIM residues that were highly conserved. The discovered compound may provide a scaffold for elaboration of other inhibitors.

## 1. Introduction

Glycolysis plays a central role in some tumor types. Many cancer cells are especially dependent on aerobic glycolysis for energy and metabolites. This dependence is known as the Warburg effect [1]. Antiglycolytic drugs acting at various steps of the glycolysis pathway have shown potential to kill or impede tumors alone or in combination with classic drugs [2–4]. To date, no TIM inhibitors suitable for targeting mammalian TIM have been reported. The cell can control glucose metabolism to some extent via TP53 [5]. In a cellular process, TP53 signaling can inhibit the Warburg effect and shift tumor glycolysis flux, converting cells to a less transformed phenotype [6]. In part this normalization is due to a shift of glucose metabolism away from glycolysis and into oxidative phosphorylation and the pentose phosphate pathways [5, 6].

TIM is a key enzyme in glycolysis catalyzing the conversion of dihydroxyacetone phosphate to glyceraldehyde-3-phosphate [7]. TIM is an essential protein, and partial function mutations in hTPI1 are incompletely tolerated in humans [8]. Deficiency phenotypes for TIM are complicated by the accumulation of its substrate, dihydroxyacetone phosphate, which is nonenzymatically converted to the toxin methylglyoxal [8]. Methylglyoxal contributes to the deleterious effects of a TIM deficiency. Speculatively, a TIM inhibitor might induce methylglyoxal production targeted to regions of high glycolysis flux, such as tumors. Potentially, this process could produce a therapeutic effect in addition to the effect of glycolysis inhibition per se.

TIM is the prototypic TIM barrel enzyme and a model for a large family of structurally related enzymes. The dihydroxyacetone-phosphate substrate binds the dimeric *α*, *β* barrel structure off-center near the internal pore. Three completely conserved residues play important catalytic roles in the TIM reaction. Glu165 abstracts a proton to begin the isomerization. This leads to formation of an enediol or enediolate stabilized by Lys13 [7]. His95 plays acid and base roles to permit resolution of the enediol. The TIM active site exhibits induced fit. A rigid loop closes over the substrate and allows movement of Glu165 into catalytic position [9]. In the closed conformation the substrate fit is very snug in the catalytic region and side reactions with water are prevented. The open conformation of TIM permits easier access to the catalytic site. The high conservation of the TIM catalytic site, and its centrality in cancer, suggests that a molecule targeted to that site might provide a therapy that could escape some drug resistance mechanisms. Compounds that inhibit TIM from trypanosomes and* Leishmania* have been found but act at a nonconserved dimer interaction site that might become mutated without loss of enzyme function [10, 11].

Structure-based screening attempts to find ligands complementary to a target binding site using a computational approach [12–15]. Generally, candidate ligands that are larger bind more tightly to a target. However, it is inefficient to thoroughly search a substantial fraction of chemical space of molecules with a drug-like (large) size distribution. Smaller fragment-sized molecules permit more thorough searches with lower numbers of molecules, but the “hits” are usually of low affinity even if they exhibit high ligand efficiency for the target site. One effective strategy is to identify small, low affinity molecules and then improve them or use them to find larger molecules.

Structure-based virtual screening involves three steps at minimum. First a molecule is docked to a protein binding site to approximate the conformation that would be achieved* in vivo* [16]. Vina is a method that is successful in this prediction [17, 18]. Second, the process is repeated over a database of diverse chemicals such as the ZINC database [19]. Finally, the docked conformers are scored to rank them according to likelihood that they would actually bind the target site. The highest ranked ligands are candidates for* in vitro* testing. Vina provides a fast score for ranking [18], but slower and more accurate molecular mechanics scoring is also an option [20, 21]. Structure-based virtual screening has been successful with many targets, for example, [12, 14, 15, 17].

The aim of this work was to discover novel TIM inhibitor(s) which might be valuable in studies of the role of glycolysis inhibition in cancer chemotherapy. We were successful in finding one compound, with a new scaffold, that may be useful by itself or allow identification of other agents of higher affinity.

## 2. Results and Discussion

### 2.1. Screen Development

The TIM binding pocket includes a catalytic site (K13, H98, E165, L230, G232), the phosphate binding site (S211, G232, N233, I170), and the contiguous pocket region (E97, L162, A163,T207,Y209, F229, V231) [7]. TIM has not been crystalized with its natural substrates. However the structure of TIM bearing substrate analogs has been solved [9, 22]. The binding region of these analogs is presumed to represent the catalytic site of TIM. The catalytic site exhibits two alternative configurations, as described, that exist in equilibrium. The closed configuration of the catalytic site (PDB:ID 1R2R chain B) is the enzymatically active form but does not allow access [9]. The open configuration (PDB:ID 1R2R chain C) is catalytically inactive but was nonetheless chosen as a ligand target, because it can accommodate a larger molecule. Blocking the open configuration should also block formation of substrate-enzyme complexes.

**Figure 1:**
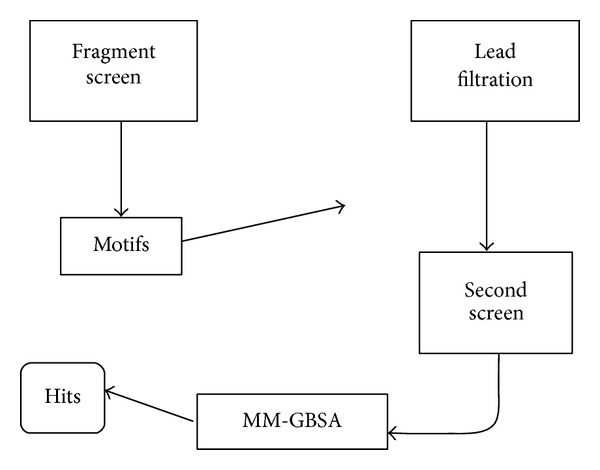
Outline of screen. Fragment screen performed first was a structure-based virtual screen on small molecules. Product of that screen was information, motifs. Filtration was based on the output of the first screen and acted on a larger, lead-like database. Filtered output was subjected to a second screen, again structure-based virtual screening. High-ranking molecules from the second screen were subjected to MM-GBSA analysis to generate a list of “hits.”

A novel strategy was developed to efficiently identify ligands that could bind this site (Figure 1). This involved a “double screen”: small molecules (fragments) were docked to TIM and common motifs of high-scoring Vina hits were identified. Docking of fragments produced a collection of hits with some common features. Fragment-based strategies have been used to predict binding motifs [23]. In many cases fragment binding is determined by biophysical methods such as X-ray crystallography [24]. Here a similar approach was employed, but the binding of fragments was examined using computational methods. Patterns that were observed included fragment binding to the TIM catalytic site, the absence of charged groups binding near the catalytic site, and presence of an amide group bound at the catalytic site. An amide moiety could be superimposed on a model of dihydroxyacetone phosphate. An amide group was also compatible with an open-configuration binding site. All high-scoring fragments showed hydrogen bonding to the catalytic Glu residue of TIM, most to the catalytic Lys as well. Almost all hits made contacts to the phosphate-binding cavity of the ligand binding site, usually with a substituted or unsubstituted benzene ring.

These criteria were used to reduce a database of lead-like (larger) compounds (338,562 total). Entries in the database inconsistent with the fragment data were eliminated using a custom Perl script. This enriched database was subjected to structure-based virtual screening, again using rabbit TIM as the target. The “double screening” approach was a computationally efficient method to screen a large chemical space. As a postscreening scoring approach, the top 5% of the high-ranked hits were visually inspected and then analyzed with a more accurate method, MM-GBSA [20, 21, 25]. The highest-ranked 7 candidates were purchased for testing* in vitro*.

### 2.2. TIM Inhibition Assay

**Figure 2:**
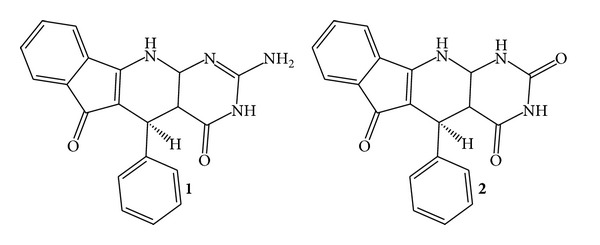
Molecules identified by screen.

**Figure 3:**
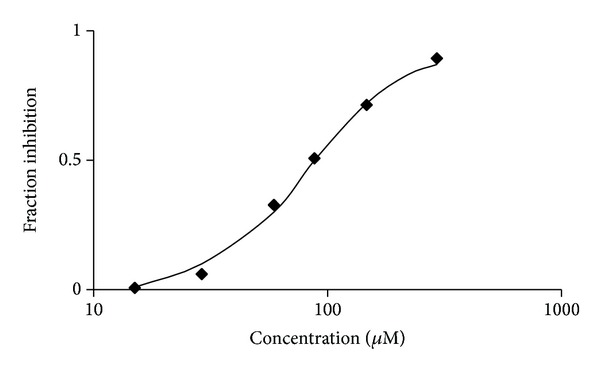
Inhibition of TIM enzyme activity by compound** 1**. Determination of enzyme activity in the presence of virtual screening hit** 1**. Assays were performed in triplicate with racemic ligand, in presence of substrate at a concentration of 0.4 mM, the *K*_*m*_.

Computational candidates were tested for the ability to inhibit the “reverse” reaction of TIM using glyceraldehyde-3-phosphate as a substrate [10]. Compound** 1** (Figure 2) was inhibited with a *K*_*i*_ of 44 *μ*M (Figure 3). Compound** 1** is a tetracyclic, with hydrophilic moieties concentrated on the pyrimidine ring, 2-amino-5-phenyl-3H-indeno[2′,1′:5,6]pyrido[2,3-d]pyrimidine-4,6(5H,11H)-dione. In general, computational methods fail to accurately predict the ranking of compound binding to a protein. However, in this case, both Vina and MM-GBSA ranked compound** 1** the highest of all tested molecules. The compound tested* in vitro* was racemic. However,** 1** was the enantiomer identified in the screen. By computational methods including MM-GBSA the other enantiomer of** 1** exhibited a low rank score for binding and is assumed to be inactive. Compound** 1** satisfied computational ADME criteria which indicate “drug-likeness” including Lipinski's “rule of five” [26] (Table 1). Preincubation of TIM with compound** 1** did not affect inhibition properties (not shown).

### 2.3. Docking Refinement

**Figure 4:**
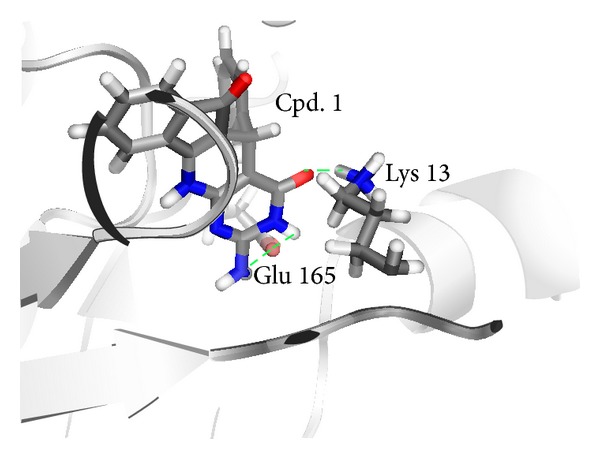
Docked conformation of ligand bound to TIM. Compound** 1** is shown as a stick representation. Side chains of TIM active site residues Lys 13 and Glu 165 are also presented as sticks. Hydrogen bonds are indicated by dashed green lines. Conformation is as determined by MD (see Section 4). A similar configuration was generated by the initial analysis with Vina docking.

**Figure 5:**
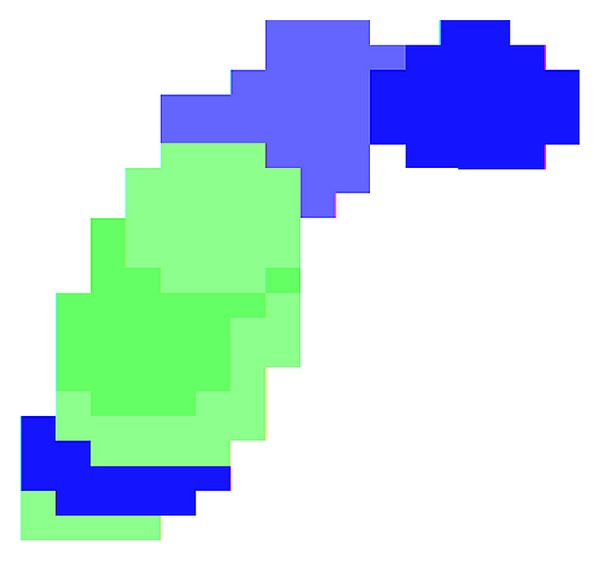
TIM binding site hydrophobicity map. The regions that a ligand encounters during binding to TIM are shown in a course-feature heatmap to emphasize broad regions of hydrophobicity and hydrophilicity in the site. Two significant hydrophobic regions and two hydrophilic regions are shown. Blue, hydrophobic; light blue, partially hydrophobic. Green, hydrophilic; light green, partially hydrophilic. The binding site was centered on binding of the substrate analog 2-phosphoglycolic acid. Flanking residues were extended manually based on topological association with the binding pocket. The lower hydrophobic region represents the active site loop region. The central hydrophilic region includes the catalytic domain and part of the phosphate-binding region. The upper hydrophobic region includes weakly hydrophobic residues that make up the bulk of the phosphate binding site and more strongly hydrophobic residues that make up the extended pocket.

To refine the ligand-enzyme complex model, 200 ps of molecular dynamics (MD) was performed. This treatment was sufficient to equilibrate side chain positions and permit minor ligand shifts without major protein structural excursions from the crystal structure. The resulting structure of the complex shows the tetracyclic ring structure occupying the cavity comprising the substrate binding region of TIM including the active catalytic site (Figures 4 and 5).

This model is supported in several ways.  Figure 5 illustrates, in a coarse-grain heatmap, the discreet hydrophobic domains of the binding pocket. Compound** 1** hydrophobicity is complementary to the hydrophobicity of pocket regions to which it is bound in Figure 4. The catalytic site to which compound** 1** is docked has catalytic residues in the dimeric configuration [27] since the coordinates were extracted from a dimeric structure for the enzyme. Thus Figure 4 may represent the conformation of an inhibitor bound to the native dimeric enzyme.

The pyrimidine ring of compound** 1** appears to hydrogen bond to catalytic residues of TIM (Lys 13, Glu 165) (Figure 4). Lys 13 forms a hydrogen bond to O1, the pyrimidine carbonyl group. Glu 165 forms a hydrogen bond to H14, of the hydrogen-bearing pyrimidine ring nitrogen. Glu 165 also bonds to an amine hydrogen. MD analysis suggests that the lysine and glutamate hydrogen bonds are stable. The catalytic site binding was consistent with the binding patterns discovered in the fragment analysis phase of this project. Compound** 2** (Figure 2) is very similar to** 1** but lacked detectable inhibitory activity (Table 1). Compound** 2**, unlike** 1**, lacks a hydrogen bond donor group at position 10 on the pyrimidine ring. Therefore** 2** may be unable to form the second hydrogen bond to Glu 165 that compound** 1** makes.

In addition to the catalytic site, per se,** 1** makes contacts with the hydrophobic lid of the catalytic site and the phosphate-binding cavity (Figures 4 and 5). The contacts to the lid are specifically to the lid in its “open” configuration, especially the contact with Ile 170. Thus** 1** may stabilize TIM in its “open” state. The contacts with the phosphate-binding cavity of TIM are hydrophobic. Part of the hydrophobic region overlaps the domain that binds the substrate phosphate (Figure 5). In crystal structures of phosphorylated substrates bound to TIM, the phosphate mostly interacts via contacts with TIM backbone atoms [9]. Some of the TIM residues that make polar contacts with the phosphate of substrate (i.e., Ser 211) interact with compound** 1** via hydrophobic portions of their side chain. In retrospective, TIM can be seen to be a moderately difficult target [28] for ligand binding. Though the catalytic site provides hydrophilic contacts, much of the binding pocket is relatively featureless. Compound** 1** makes a good van der Waals fit, however, with 26 atoms contacting the enzyme.

### 2.4. Conservation of Ligand Contacts

One serious problem with anticancer agents is the development of drug resistance. One mechanism of resistance involves mutation of the target protein to lose binding. Compound** 1** was selected to bind to a conserved region of TIM that might not mutate without loss of function. Contacts of** 1** were mapped to the sequence of TIM to determine if the contacts were, in fact, to highly conserved residues that might not mutate without loss of function.

#### 2.4.1. Conservation of Inhibitor-Contacted Residues of TIM

*Contacted Clusters. *All of these residues were conserved in 6 vertebrate species: cow, rat, dog, chimpanzee, chicken, and zebrafish:

- catalytic region residues: N11, K13, H95, E165, P166;
- active-site loop: I170;
- phosphate-binding site: G209, G210, S211, V212;
- extended pocket: L230, G232, G233, A234.

This shows the contacts made by** 1**, all of which represented TIM residues that were completely conserved in representative mammals and other vertebrates. Thus compound** 1** might interact only with residues required for TIM function and thus avoid drug resistance during therapy due to mutation of the target to a nonbinding, but active, state.

## 3. Conclusions

Structure-based virtual screening was used to identify an inhibitor of TIM. The modifications we developed to efficiently carry out this screen may be useful as well to others to reduce the computational expense of virtual screening. The inhibitor we found represents a possible scaffold for a novel class of antiglycolytic inhibitors targeting TIM. This inhibitor has prominent interactions with the catalytic site of TIM and interacts as well over the entire ligand binding site of TIM. This type of molecule might have the potential to inhibit or reverse aerobic glycolysis in cancer, an intriguing, but still unproven, approach to cancer chemotherapy. The binding envelope for the compound we found is completely conserved suggesting that TIM might not mutate to resistance. Thus the discovered compound, and analogs, could be useful exploratory additions to the known inhibitors of other glycolytic enzymes.

## 4. Materials and Methods

### 4.1. Database of Ligands

The ZINC database [19] subsets for fragments and lead-like compounds were screened. All molecules were computationally equilibrated at pH 7.0. In cases where candidate structures were ambiguous, alternative charged forms or tautomers were tested as well as the original database entry. The ZINC code for compound** 1** is Zinc-04384801. The ZINC codes for the other purchased compounds were 00087820, 19169090, 23625983, 11009166, 65498992, and 14981986.

### 4.2. Chemicals

Reagents were purchased from Sigma-Aldrich Chemical Corp. Candidate small-molecule inhibitors were purchased from Molport Inc., dissolved in DMSO, and stored at −80°C.

### 4.3. Virtual Screen for TIM Inhibitors

The first stage of the virtual screen focused on ligand fragments averaging 221 MW; the second targeted lead-like molecules with a MW averaging 296. Vina [18] was used as the docking software. Structure-based virtual screening used PDB:ID 1R2R chain C (http://www.pdb.org/pdb/), rabbit TIM as a target [9]. PDB:ID 1R2R chain A performed less well, apparently because of the side chain conformation of Ile 170 of the active site loop. Docking scores for ZINC database fragments with this structure yielded lower overall scores. Docking of 37,647 fragments from the ZINC database [19] with PDB:ID 1R2R chain C yielded 22 modestly high-scoring hits which were analyzed for common motifs.

The motif criteria (an amide moiety) were used to screen a database of 338,562 lead-like molecules using a custom Perl script to filter the ZINC database. Approximately 10% of the lead-like molecules exhibited features compatible with the putative criteria for high-affinity binding and were retained for the next phase of virtual screening. After the second round of virtual screening, postscreening with MM-GBSA scoring [20, 21, 25] and visual inspection further reduced the number of candidate ligands.

### 4.4. MM-GBSA Ranking

Vina-generated complexes were minimized using implicit water (generalized Born model) in the Amber 9.0 package. The structures were submitted to 300 cycles of steepest descent and 700 cycles of conjugant gradient minimization. Amber MM-GBSA was performed on minimized structures without MD simulation [25].

### 4.5. Enzyme Assays

The standard TIM coupled assay [10] contained glyceraldehyde-3-phosphate (0.4 mM), NADH (0.1 mM), *α*-glycerophosphate dehydrogenase (0.5 units/mL), and triosephosphate isomerase (rabbit muscle TIM) (0.01 units/mL) in 0.1 M triethanolamine hydrochloride buffer, pH 8.0 [10]. The reaction was initiated by addition of TIM and followed at 340 nm. Candidate inhibitors were added as DMSO solutions; vehicle alone was added to controls. *K*_*i*_ was calculated as *K*_*i*_ = *K*_app_/(1 + [S]/*K*_*m*_), where *K*_app_ is the apparent dissociation constant (IC50), [S] is the substrate (glyceraldehyde-3-phosphate) concentration, and *K*_*m*_ is the Michaelis constant for TIM (0.4 mM glyceraldehyde-3-phosphate). Initial screening was at 300 *μ*M of the test substance.

### 4.6. Molecular Dynamics

The 1R2R chain C/compound** 1** complex produced by Vina docking was subjected to a brief MD simulation using the Amber force fields with explicit water [20, 21]. The complex was placed in an octagonal box with 5749 molecules of water. The docked structure of** 1** with TIM was relaxed with 200 cycles of steepest descent minimization and 300 cycles of conjugant gradient minimization. Molecular dynamics simulation was carried out with protein backbone restraints to speed equilibration and SHAKE. Ligand was not restrained. After a short equilibration, the complex was subjected to 200 ps molecular dynamics using the Amber force fields ff99 and gaff. The intent was to allow side chains of residues to partially equilibrate without allowing the backbone of TIM to significantly realign. The final snapshot of the trajectory was used for visualization. The stability of intermolecular contacts in the complex was confirmed by analysis of the entire trajectory with the MD visualization tool VMD. The time frame of the simulation only allowed observation of side chain movements.

### 4.7. Hydrophobicity

To identify binding features of the TIM binding pocket, hydrophobicity was determined using the Kyte and Doolittle scale [29]. Categories from hydrophobic to hydrophilic were >2.0, 2.0–0, −2.0–0, <−2.0. A 2D heatmap (Figure 5) was prepared including only the 21 binding pocket residues of TIM to clarify feature domains.

### 4.8. Binding Site Conservation

TIM protein sequences of diverse organisms were retrieved from NCBI (http://www.ncbi.nlm.nih.gov/entrez/). Sequences were aligned using ClustalW [30]. The TIM residues contacted by compound** 1** docked as above (approximation of 3.6 Angstroms or less between ligand and protein) were mapped to the sequence alignment and the conservation status of each contacting residue was determined. Contacting residues for which the sequence was unchanged for the entire series of vertebrate proteins were classified as conserved. Conserved sites may play an essential role in some aspect of TIM function.

### 4.9. ADME Properties

Compound** 1** was assessed using Lipinski's “Rule of Five” criteria [26] (Table 1). This method assesses similarity of properties of candidate drugs to those of approved drugs. The quantity xLogP (Table 1), calculated by the ZINC curators [19], was used instead of cLogP used by Lipinski for calculating hydrophobicity (both measures are similar computational indicators of lipophilicity).

## Figures and Tables

**Table 1:** Properties of compounds.

Compound	1	2
*K*_*i*_ (TIM) (*μ*M)	44	>440
MW	342	343
HB donors	4	3
HB acceptors	6	6
*x*log⁡*P*	2.95	2.79
